# Logarithmic Scaling of Loss Functions for Enhanced Self-Supervised Accelerated MRI Reconstruction

**DOI:** 10.3390/diagnostics15232993

**Published:** 2025-11-25

**Authors:** Jaejin Cho

**Affiliations:** Department of Artificial Intelligence and Robotics, Sejong University, Seoul 05006, Republic of Korea; jaejincho@sejong.ac.kr

**Keywords:** deep-learning-based image reconstruction, magnetic resonance imaging, logarithm-scaled loss, scan-specific MRI reconstruction, self-supervised learning

## Abstract

**Background/Objectives:** Magnetic resonance imaging (MRI) is a widely used non-invasive imaging modality that provides high-fidelity soft-tissue contrast without ionizing radiation. However, acquiring high-resolution MRI scans is time-consuming, necessitating accelerated acquisition and reconstruction methods. Recently, self-supervised learning approaches have been introduced for reconstructing undersampled MRI data without external fully sampled ground truth. **Methods:** In this work, we propose a logarithmic scaled scheme for conventional loss functions (e.g., $\ell_{1}$ , $\ell_{2}$ ) to enhance self-supervised MRI reconstruction. Standard self-supervised methods typically compute loss in the *k*-space domain, which tends to overemphasize low spatial frequencies while under-representing high-frequency information. Our method introduces a logarithmic scaling to adaptively rescale residuals, emphasizing high-frequency contributions and improving perceptual quality. **Results:** Experiments on public datasets demonstrate consistent quantitative improvements when the proposed log-scaled loss is applied within a self-supervised MRI reconstruction framework. **Conclusions:** The proposed approach improves reconstruction fidelity and perceptual quality while remaining lightweight, architecture-agnostic, and readily integrable into existing self-supervised MRI reconstruction pipelines.

## 1. Introduction

Magnetic resonance imaging (MRI) is a non-invasive imaging modality that provides excellent soft-tissue contrast, making it essential for neurological, musculoskeletal, and cardiovascular diagnostics [1]. Unlike X-ray or computed tomography (CT), MRI does not rely on ionizing radiation and offers versatile contrast mechanisms through parameter-controlled pulse sequences. However, its inherently slow data acquisition, particularly in high-resolution, multi-contrast, or multi-directional protocols, limits clinical throughput and increases susceptibility to motion artifacts [2]. Prolonged scan times not only compromise patient comfort but also elevate the likelihood of motion-induced data corruption, thereby motivating the development of accelerated acquisition and reconstruction techniques.

Traditional parallel imaging (PI) methods, such as sensitivity encoding (SENSE) [3] and generalized autocalibrating partially parallel acquisition (GRAPPA) [4], have long served as foundational frameworks for accelerating MRI acquisition. These methods exploit the distinct spatial sensitivity profiles of multi-channel receiver coils to reconstruct images from undersampled measurements in the spatial frequency domain (*k*-space). Although PI techniques are widely implemented in clinical MRI systems and support moderate acceleration rates, their performance is inherently constrained by coil geometry and degradation of the signal-to-noise ratio (SNR). As the acceleration factor increases, PI reconstructions become increasingly prone to aliasing artifacts and amplified noise.

To overcome the limitations of PI, model-based approaches [5] such as compressed sensing (CS) [6,7] and low-rank matrix recovery [8,9] have emerged as powerful alternatives. CS exploits the fact that MR images are sparse or compressible in certain transform domains such as wavelet and total variation, enabling accurate reconstruction from substantially fewer measurements than those required by the Nyquist criterion. Low-rank methods, on the other hand, exploit redundancies across *k*-space [8], temporal frames [10], or coil channels [11] to impose structural priors. These techniques have proven to be effective, particularly in dynamic MRI and quantitative imaging applications. However, they typically involve iterative optimization procedures and require careful hyperparameter tuning; otherwise, they may produce over-smoothed results or structural artifacts. Furthermore, their reliance on hand-crafted priors limits adaptability to complex anatomical variations and scanner-dependent noise characteristics.

In recent years, deep learning has emerged as a powerful data-driven alternative for accelerated MRI reconstruction [12]. Neural networks trained on large datasets can learn expressive representations of MR signals and directly map undersampled measurements to high-quality reconstructions. Architectures such as U-Nets [13], variational networks [14], and unrolled optimization frameworks [15] have demonstrated state-of-the-art performance in various MRI applications. These models enable faster inference and improved generalization across diverse anatomical regions. Among them, model-based deep learning (MoDL) [15] integrates residual networks within a physics-constrained optimization framework, achieving high reconstruction quality with relatively few trainable parameters. Nevertheless, the success of these supervised methods strongly depends on large-scale datasets with fully sampled ground-truth references, which are expensive and time-consuming to acquire in practice.

Self-supervised MRI reconstruction methods overcome the limitations of supervised learning by defining loss functions directly from the available undersampled measurements [16,17,18]. A common strategy is to divide the acquired *k*-space data into disjoint subsets, using one for network input and another to enforce data consistency. Recent advances, such as zero-shot self-supervised learning (ZS-SSL) [19,20], further extend this concept by partitioning the *k*-space into three distinct sets: one for training input, one for computing the loss, and one for validating the loss during training. This approach eliminates the need for fully sampled ground-truth data while achieving reconstruction performance comparable to that of supervised learning, thereby enabling broader applicability in clinical MRI.

Building upon ZS-SSL, we recently introduced the zero-shot self-supervised learning of multi-shot image reconstruction for improved diffusion MRI (zero-MIRID) framework [21], which extends self-supervised learning to diffusion MRI (dMRI). In dMRI, acquiring fully sampled diffusion-weighted data across all diffusion directions and b-values is infeasible in clinical settings due to prolonged scan times and motion sensitivity. This limitation makes large-scale supervised learning for dMRI reconstruction impractical and further underscores the importance of self-supervised approaches in this domain.

Zero-MIRID incorporates a dual-domain residual network that operates in both the image and *k*-space domains, enabling the model to leverage complementary information from each domain. Previous studies have demonstrated that utilizing both domains can improve the fidelity of the final reconstructed images [22,23]. To further enhance performance, particularly for echo-planar imaging (EPI) sequences that employ partial Fourier (PF) acquisition, the virtual coil technique was adopted to exploit the conjugate symmetry of *k*-space [24].

Despite these advances, most self-supervised methods compute loss values in the *k*-space domain [19,20], where low spatial frequencies dominate the signal energy spectrum. This imbalance inherently biases the learning process toward low-frequency components, often at the expense of high-frequency details that are essential for preserving edges and subtle anatomical structures.

In this study, we propose a logarithmic scaling scheme applied to conventional loss functions such as $\ell_{1}$ and $\ell_{2}$ to mitigate this frequency bias. It is also worth noting that the MRI forward model measures the Fourier transform of the object using complex exponential bases, $e^{-i2\pi kx}$ . Because the energy of *k*-space coefficients spans several orders of magnitude under these exponential bases, residuals in the Fourier domain can be highly unbalanced across frequencies. Applying a logarithmic transformation to the residual magnitude provides a natural dynamic-range compression that aligns with the exponential formulation of the Fourier transform, resulting in more balanced gradient contributions from both low- and high-frequency components. This simple yet effective modification enables the network to better capture fine structural details while preserving the self-supervised nature of the learning process. We apply the proposed log-scaled loss to the zero-MIRID framework for conventional voxel-contrast images, including $T_{1}$ - and $T_{2}$ -weighted MR images. Furthermore, we explore the potential of extending zero-MIRID, originally developed for diffusion MRI (dMRI), to other voxel-contrast imaging modalities.

To the best of our knowledge, this is the first study to incorporate logarithmic scaling into loss functions for self-supervised MRI reconstruction. By directly addressing the frequency imbalance inherent in *k*-space–based training, the proposed method enhances the perceptual fidelity of reconstructed images without requiring any architectural modifications or fully sampled ground-truth data.

## 2. Theory

### 2.1. Parallel Imaging Problem

In parallel MRI, multiple receiver coils with distinct spatial sensitivity profiles are used to accelerate image acquisition by undersampling the *k*-space. Let $x\in C^{N}$ denote the complex-valued target image to be reconstructed, and let $S_{c}\in C^{N}$ represent the spatial sensitivity map of the *c*-th coil for c=1,…,C . The fully sampled *k*-space measurement for each coil is modeled as

(1) $$y_{c}=FS_{c}x+\epsilon_{c},$$

where F denotes the 2D Fourier transform operator and $\epsilon_{c}$ is measurement noise. Note that $S_{c}$ and F are treated as linear operators, and $FS_{c}x$ represents the sequential application of these operators to *x*.

To accelerate the scan, *k*-space is sampled at a reduced set of positions Ω . The corresponding undersampled measurements are expressed as

(2) $$y_{c,\Omega }=P_{\Omega }FS_{c}x+\epsilon_{c},$$

where $P_{\Omega }$ is a binary sampling operator that masks the acquired *k*-space locations.

In the image domain, undersampling introduces aliasing due to violation of the Nyquist criterion. SENSE formulates the reconstruction as an inverse problem using the estimated coil sensitivities:

(3) $$x=\underset{x}{\mathrm{argmin}}{∥P_{\Omega }FSx-y_{\Omega }∥}_{2}^{2},$$

where $y_{\Omega }\in C^{N}$ denotes the undersampled measurements in stacked form from all coils and *S* is the combined sensitivity operator.

For further improved image reconstruction, the low-rank constraints, such as structured low-rank constraint with smooth phase prior (S-LORAKS) [25], leverage the low-rank structure of the interface, the conjugate symmetry of the *k*-space, and phase consistency. The image reconstruction problem is formulated as

(4) $$x=\underset{x}{\mathrm{argmin}}{∥P_{\Omega }FSx-y_{\Omega }∥}_{2}^{2}+\lambda J\left(Fx\right),$$

where J denotes a low-rank regularization term in the *k*-space domain, and λ controls the regularization strength.

### 2.2. Deep Learning-Based MRI Reconstruction

Although conventional PI methods such as SENSE typically offer 2–3-fold acceleration in head imaging, their performance deteriorates at higher acceleration rates due to noise amplification and residual aliasing. Deep learning-based approaches have emerged as powerful alternatives by learning data-driven image priors from large-scale training data.

MoDL integrates neural networks into traditional optimization frameworks [15]. MoDL unrolls an iterative reconstruction scheme alternating between data consistency (DC) and learned denoising as follows:

(5) $$x=\underset{x}{\mathrm{argmin}}{∥P_{\Omega }FSx-y_{\Omega }∥}_{2}^{2}+\lambda {∥x-R_{\theta }\left(x\right)∥}_{2}^{2},$$

where $R_{\theta }$ is a CNN trained to reduce artifacts.

Inspired by MoDL and related frameworks such as MoDL-MUSSELS [23] and KIKI-net [22], zero-MIRID further incorporates a residual CNN in the *k*-space domain alongside the image-domain denoiser. Additionally, a virtual coil operator V is used to exploit conjugate symmetry in the partially sampled *k*-space. The zero-MIRID formulation is given by

(6) $$x=\underset{x}{\mathrm{argmin}}{∥P_{\Omega }FSx-y_{\Omega }∥}_{2}^{2}+\lambda_{1}{∥V^{H}N_{i}Vx∥}_{2}^{2}+\lambda_{2}{∥V^{H}F^{H}N_{k}FVx∥}_{2}^{2},$$

where $N_{i}$ and $N_{k}$ are residual denoisers operating in the image and *k*-space domains, respectively, and $\lambda_{1}$ and $\lambda_{2}$ denote trainable regularization weights for the image- and *k*-space-domain denoisers, respectively. The operators V and $V^{H}$ denote the virtual coil projection and its Hermitian transpose.

Despite their success, most deep learning models rely on supervised learning with fully sampled ground truth, which is impractical for many clinical protocols including DWI.

### 2.3. Self-Supervised Learning for MRI Reconstruction

To avoid the need for fully sampled reference data, self-supervised learning strategies have been proposed. ZS-SSL partitions the acquired *k*-space indices Ω into three disjoint subsets: Λ , Θ , and Ψ . During training, Λ is used as network input and Θ to compute the loss:

(7) $$L\left(P_{\Theta }y,P_{\Theta }FSf\left(P_{\Lambda }y;\theta \right)\right),$$

where f(·;θ) is the learned reconstruction network. In the validation phase, Λ∪Θ is used as input and Ψ is held out for loss evaluation:

(8) $$L\left(P_{\Psi }y,P_{\Psi }FSf\left(P_{\Lambda \cup \Theta }y;\theta \right)\right).$$

In inference time, the entire measurement set Ω is used. In our experiments, we use the combination of the normalized root mean square error (NRMSE) for $\ell_{2}$ loss and the normalized mean absolute error (NMAE) for $\ell_{1}$ loss.

Despite their effectiveness, these methods typically compute the loss in the *k*-space domain, where the signal energy is dominated by low spatial frequencies. This introduces a training bias that under-represents high-frequency features crucial for preserving edges and fine detail.

### 2.4. Logarithmic Scaling of the Loss

To mitigate this frequency bias, we propose a logarithmic weighting scheme that rebalances the loss toward high-frequency components. We explore a log-scaled residual loss, formulated as

(9) $$\log(1+|PFS\tilde{x}-Py|).$$

The scaled loss emphasizes high-frequency residuals during training, helping preserve structural details without altering network architectures or training pipelines. Our approach is lightweight, generalizable, and compatible with existing self-supervised frameworks.

We also utilized the combination of the NRMSE for $\ell_{2}$ loss and the NMAE for $\ell_{1}$ loss to train and validate the network. Specifically, the log-scaled loss values can be described as follows:

(10) $$L_{p}\;=\;\frac{∥\log\;\left(1+|\hat{y}-y|\right){∥}_{p}^{p}}{∥\log\;\left(1+|y|\right){∥}_{p}^{p}},\;p\in \left\{1,2\right\},$$

where $\hat{y}$ and *y* denote the reconstructed and measured *k*-space data, respectively, and ${∥\cdot ∥}_{p}$ represents the *p*-norm. This formulation ensures that the $\ell_{1}$ and $\ell_{2}$ losses are scale-normalized, yielding a scale-invariant range (0–100%) comparable across different sampling patterns and data magnitudes.

## 3. Materials and Methods

### 3.1. Network Architecture

**Figure 1:**
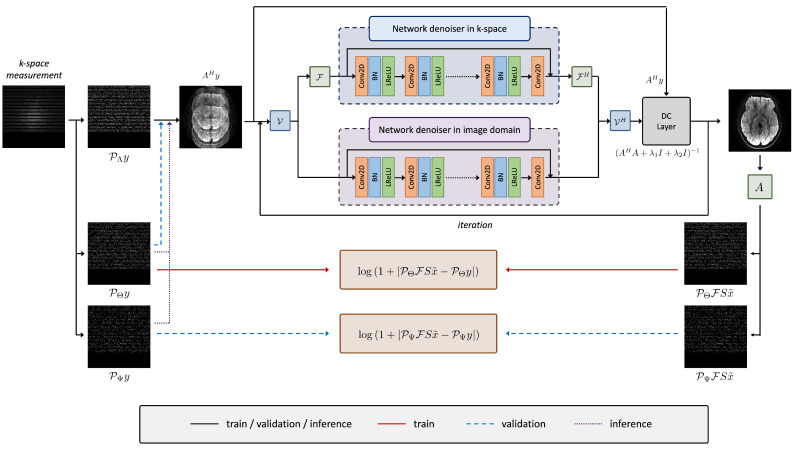
Zero-MIRID network architecture used for MR image reconstruction. The acquired *k*-space data were divided into three disjoint subsets for self-supervised learning. The red line indicates the logarithmic loss used for training, whereas the blue lines denote the validation loss. All subsets were utilized for final image reconstruction during inference.

Figure 1 illustrates the zero-MIRID network architecture [21], which was employed for image reconstruction in this study. The network input is defined as $A^{H}y$ , where A=PFS represents the forward operator composed of the sampling mask P , the Fourier transform F , and the coil sensitivity operator *S*. The architecture consists of two convolutional neural networks (CNNs) operating in the *k*-space and image-space domains, respectively. Virtual coil augmentation [24] is applied before each denoising CNN and subsequently removed to exploit the conjugate symmetry of partially acquired *k*-space data.

The reconstruction formulation follows Equation (6). We adapt the alternating minimization framework originally proposed in MoDL [15] to solve Equation (6). The iterative update scheme is defined as

(11) $$\begin{array}{cc} x_{n+1} & ={\left(A^{H}A+\lambda_{1}I+\lambda_{2}I\right)}^{-1}\left(A^{H}y+\lambda_{1}\eta_{n}+\lambda_{2}\zeta_{n}\right),\\ \zeta_{n+1} & =VF^{H}R_{\theta ,k}FVx_{n+1},\\ \eta_{n+1} & =V^{H}R_{\theta ,i}Vx_{n+1}, \end{array}$$

where *n* denotes the iteration index, and $\eta_{n}$ and $\zeta_{n}$ represent the outputs of the denoising networks $R_{\theta ,i}$ and $R_{\theta ,k}$ operating in the image and *k*-space domains, respectively.

### 3.2. Experiment Details

SENSE reconstructions were implemented in MATLAB R2022a and executed on an AMD Ryzen 9 9900X CPU with 128 GB of RAM. All neural network experiments were conducted in Python 3.10 using the Keras API of TensorFlow 2.17.0 and trained on an NVIDIA H100 GPU with 94 GB of memory.

Each denoising CNN consisted of 15 convolutional layers with a kernel size of 3×3 . For zero-MIRID, each layer contained 46 channels, whereas 64 channels per layer were used for ZS-SSL to match the number of trainable parameters (approximately $5.41\times 10^{5}$ and $5.20\times 10^{5}$ , respectively). The data-consistency (DC) module employed ten conjugate-gradient iterations, and the entire reconstruction block was unrolled ten times. Leaky ReLU was used as the activation function, and the Adam optimizer was applied with a learning rate of $10^{-3}$ .

For each slice, one validation subset Ψ and 50 unique training subsets (Λ,Θ) were randomly generated. The ratio of *k*-space points assigned to Λ : Θ : Ψ was 0.48 : 0.32 : 0.20 . A single network was trained and subsequently used for reconstruction of all slices. To train and validate the network, we employed the normalized $\ell_{1}$ and $\ell_{2}$ losses, the proposed log-scaled $\ell_{1}$ and $\ell_{2}$ losses, and their combined forms.

All experiments used the multi-channel brain dataset from the publicly available fastMRI dataset [26]. For scan-specific MR imaging, data from one subject were selected for both 1D and 2D subsampling experiments to train and evaluate the neural networks with the proposed log-scaled loss functions.

#### 3.2.1. 1D Subsampling

We retrospectively subsampled $T_{2}$ -weighted MR images acquired with a 20-channel head coil from one subject, consisting of 16 slices. Subsampling was performed along a single phase-encoding direction, as in conventional two-dimensional (2D) multi-slice MRI acquisitions. Each slice was accelerated by a factor of five ( R=5 ) using a 6/8 PF acquisition. A single network was trained across all slices.

#### 3.2.2. 2D Subsampling

We retrospectively subsampled post-contrast $T_{1}$ -weighted MR images acquired with a 20-channel head coil from one subject, consisting of 16 slices. Subsampling was performed in both phase-encoding directions using a two-dimensional Controlled Aliasing in Parallel Imaging (CAIPI) strategy for 3D MRI acquisitions [27]. Each slice was accelerated by a factor of nine ( R=3×3 ) using a 7/8 PF acquisition. A single network was trained across all slices.

## 4. Results and Discussion

### 4.1. 1D Subsampling

**Figure 2:**
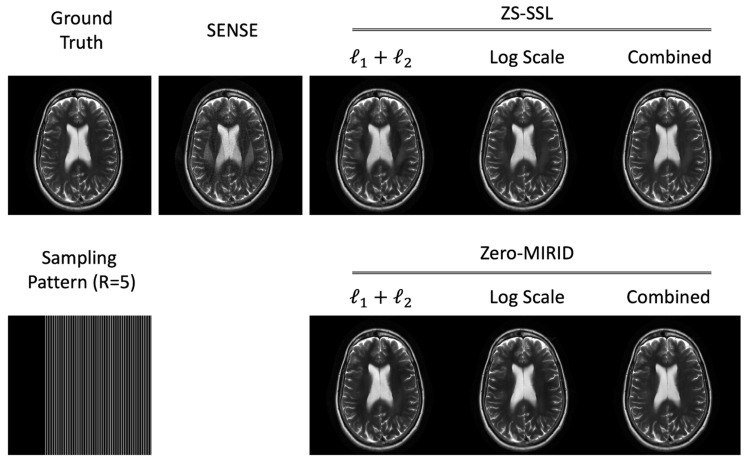
Reconstructed images from 1D subsampled data. The *k*-space data were retrospectively subsampled ( R=5 ) using a 6/8 PF acquisition, as illustrated in the sampling pattern.

Figure 2 presents reconstructed images obtained with SENSE, ZS-SSL, and zero-MIRID. For ZS-SSL and zero-MIRID, we evaluated three loss configurations: conventional normalized $\ell_{1}$ and $\ell_{2}$ , the proposed log-scaled $\ell_{1}$ and $\ell_{2}$ , and the combined conventional and log-scaled losses. SENSE reconstructions exhibited residual folding artifacts and noise amplification. ZS-SSL produced smoother images but still contained unresolved folding artifacts. ZS-SSL trained with the log-scaled and combined losses yielded better-resolved images, although minor folding artifacts remained. In contrast, zero-MIRID substantially reduced folding artifacts across all loss configurations, with the log-scaled and combined losses demonstrating superior image quality compared with the conventional loss.

Table 1 summarizes quantitative metrics corresponding to the reconstructions in Figure 2. We evaluated the normalized root mean square error (NRMSE), peak signal-to-noise ratio (PSNR), structural similarity index measure (SSIM) [28], feature similarity index (FSIM) [29], high-frequency error norm (HFEN), learned perceptual image patch similarity (LPIPS) [30], and gradient magnitude similarity deviation (GMSD) [31]. When trained with conventional losses, ZS-SSL outperformed SENSE, while zero-MIRID further improved all metrics. ZS-SSL with log-scaled losses achieved substantially better scores than with conventional losses, and the combined loss offered additional improvement. For zero-MIRID, the log-scaled loss enhanced all quantitative measures, whereas the combined loss yielded comparable results relative to the log-scaled case.

### 4.2. 2D Subsampling

**Figure 3:**
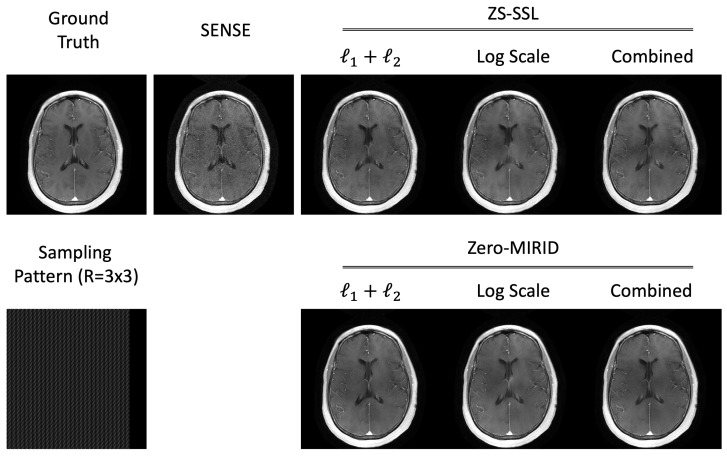
Reconstructed images from 2D subsampled data. The *k*-space data were retrospectively subsampled ( R=3×3 ) using a 7/8 PF acquisition, as illustrated in the sampling pattern.

Figure 3 presents reconstructed images obtained with SENSE, ZS-SSL, and zero-MIRID. For ZS-SSL and zero-MIRID, we evaluated three loss configurations: conventional normalized $\ell_{1}$ and $\ell_{2}$ , the proposed log-scaled $\ell_{1}$ and $\ell_{2}$ , and the combined conventional and log-scaled losses. SENSE reconstructions exhibited residual folding artifacts and noticeable noise amplification. ZS-SSL produced smoother images with fewer visible artifacts, although some residual aliasing remained in some configurations. In contrast, zero-MIRID further mitigated noise and aliasing compared with both SENSE and ZS-SSL.

Table 2 summarizes the quantitative metrics corresponding to the reconstructions in Figure 3. The evaluation included NRMSE, PSNR, SSIM, FSIM, HFEN, LPIPS, and GMSD. Among the methods trained with conventional losses, SENSE produced the lowest scores across all metrics. ZS-SSL achieved better NRMSE, PSNR, and HFEN values, whereas zero-MIRID achieved higher SSIM, FSIM, LPIPS, and GMSD scores compared with each other. When using the proposed log-scaled or combined losses, both ZS-SSL and zero-MIRID exhibited improved performance across most metrics compared with their conventional counterparts.

### 4.3. Residual Error Analysis

**Figure 4:**
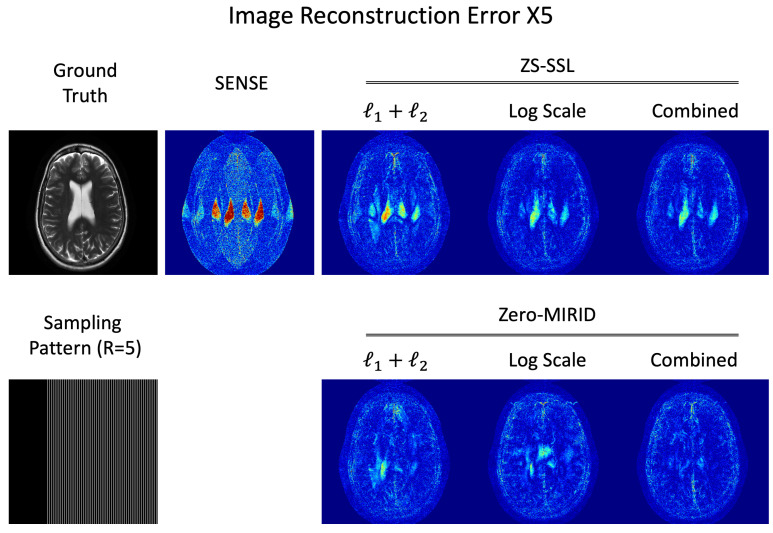
Residual error maps from 1D subsampled data ( R=5 , PF = 6/8).

Figure 4 illustrates the residual error maps from image reconstructions in the 1D subsampling experiment. As summarized in Table 1, ZS-SSL trained with the combination of the conventional and proposed log-scaled loss functions achieved the best performance across five quantitative metrics. However, the residual error maps reveal that some slices still exhibit severe unfolding artifacts that could not be fully mitigated even with ZS-SSL. In contrast, zero-MIRID produced substantially lower residual errors, particularly in regions affected by folding artifacts.

**Figure 5:**
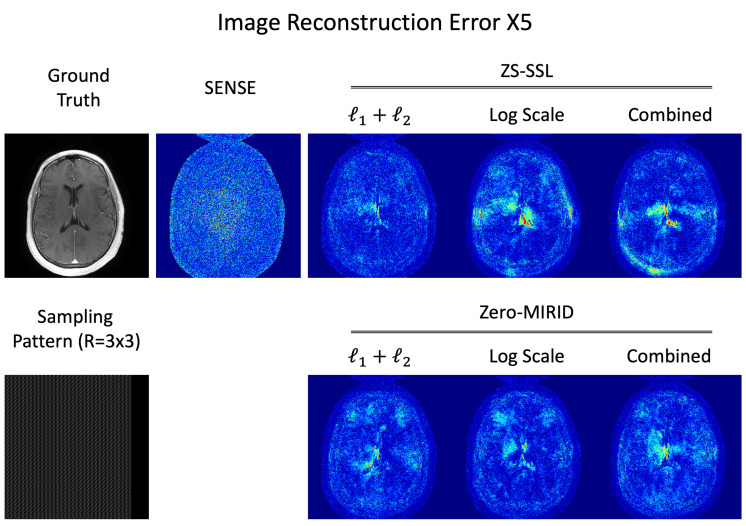
Residual error maps from 2D subsampled data ( R=3×3 , PF = 7/8).

Figure 5 illustrates the residual error maps from image reconstructions in the 2D subsampling experiment. As summarized in Table 2, zero-MIRID trained with the proposed log-scaled loss functions achieved the best performance across most quantitative metrics. The residual error maps consistently show fewer artifacts in zero-MIRID with the proposed loss, in agreement with the quantitative evaluations. Interestingly, ZS-SSL trained with the conventional loss yielded the best HFEN values, which is also visually reflected in the residual error maps.

### 4.4. Key Findings and Analysis

According to the quantitative metrics, the combined loss, comprising the conventional and log-scaled terms, achieved performance comparable to, and in some cases superior to, that of the log-scaled loss alone. These findings suggest that the logarithmic and conventional losses are not mutually exclusive but can be effectively integrated to further enhance reconstruction performance. Although the log-scaled loss improves reconstructed image quality, excessively strong logarithmic scaling may reduce convergence stability when large residuals are present. Therefore, combining the conventional and log-scaled losses helps stabilize network training while maintaining high-frequency fidelity.

When comparing the two frameworks under the conventional loss configuration, zero-MIRID consistently outperformed ZS-SSL, except for the HFEN metric in the 2D subsampling experiment. With log-scaled losses, ZS-SSL exhibited the most substantial performance improvement under the 1D subsampling condition, highlighting the benefit of frequency reweighting in single-direction undersampling scenarios. In both frameworks, incorporating the log-scaled loss improved all quantitative results relative to conventional loss functions.

To provide a practical assessment of computational efficiency, we measured the wall-clock inference time per subject for all reconstruction methods on the same NVIDIA H100 GPU. SENSE required approximately 620 ms per subject, ZS-SSL required 850 ms, and zero-MIRID required 1250 ms. The additional computation in zero-MIRID arises from its dual-domain CNN structure and the virtual-coil augmentation steps, which introduce more convolutions and operator applications than in ZS-SSL. Importantly, the proposed log-scaled loss does not introduce any additional inference-time cost, as it affects only the training objective and does not modify the network architecture.

In this study, a single network was trained across all slices and used for inference in a subject. Slight variations in performance were observed across slices, which may be attributed to the limited training data relative to the network capacity. Training with a larger dataset including multiple subjects in a self-supervised manner may yield more generalized networks and enable more stable and comprehensive performance analysis. Overall, the experimental findings consistently demonstrate that the proposed logarithmic scaling improves reconstruction fidelity by balancing frequency contributions during training.

## 5. Conclusions

This study proposed a logarithmically scaled loss function to enhance MR image reconstruction. Integrating the proposed loss into self-supervised frameworks consistently improved image quality and structural fidelity, as confirmed by quantitative metrics. The log-scaled loss was validated in two scenarios, 1D subsampling for 2D MR scans and 2D-CAIPI subsampling for 3D acquisitions, both demonstrating superior performance over conventional losses in ZS-SSL and zero-MIRID frameworks.

Future work will investigate alternative undersampling strategies such as Poisson-disc or variable-density random sampling [6,7]. In addition, the proposed log-scaled loss will be evaluated on larger datasets, diverse MR sequences, and different network architectures to further assess its robustness and generalizability. Furthermore, we plan to extend the experiments to multiple subjects and incorporate cross-validation analyses to more comprehensively evaluate the generalization capability of the proposed framework across varying anatomical and acquisition conditions.

## Figures and Tables

**Table 1:** Quantitative evaluation metrics for 1D subsampling. Note that ↑ indicates higher is better and ↓ indicates lower is better in each metric.

	SENSE	ZS-SSL			Zero-MIRID		
	-	$\ell_{1}+\ell_{2}$	$\ell_{1}+\ell_{2}$ Log Scale	Combined	$\ell_{1}+\ell_{2}$	$\ell_{1}+\ell_{2}$ Log Scale	Combined
**NRMSE** (↓)	13.74	10.28	7.58	**7.47**	8.83	8.84	8.36
**PSNR** (↑)	29.80	32.32	34.96	**35.08**	33.64	33.63	34.11
**SSIM** (↑)	0.8585	0.9154	0.9477	0.9485	0.9302	**0.9496**	0.9465
**FSIM** (↑)	0.9683	0.9848	0.9889	**0.9891**	0.9878	0.9884	0.9878
**HFEN** (↓)	0.1571	0.1185	0.0765	**0.0700**	0.0843	0.1017	0.0879
**LPIPS** (↓)	0.1229	0.0810	0.0602	**0.0588**	0.0684	0.0599	0.0596
**GMSD** (↓)	0.1932	0.1525	0.1328	0.1321	0.1428	**0.1310**	0.1332

↑: higher values indicate better performance; ↓: lower values indicate better performance.

**Table 2:** Quantitative evaluation metrics for 2D subsampling. Note that ↑ indicates higher is better and ↓ indicates lower is better in each metric.

	SENSE	ZS-SSL			Zero-MIRID		
	-	$\ell_{1}+\ell_{2}$	$\ell_{1}+\ell_{2}$ Log Scale	Combined	$\ell_{1}+\ell_{2}$	$\ell_{1}+\ell_{2}$ Log Scale	Combined
**NRMSE** (↓)	11.78	6.77	7.64	7.32	7.81	**6.66**	7.65
**PSNR** (↑)	31.71	36.52	35.47	35.83	35.28	**36.65**	35.46
**SSIM** (↑)	0.7896	0.9288	0.9472	0.9470	0.9421	0.9485	**0.9508**
**FSIM** (↑)	0.9637	0.9929	0.9934	0.9935	0.9939	**0.9942**	0.9940
**HFEN** (↓)	0.1107	**0.0731**	0.0994	0.0882	0.0885	0.0882	0.0836
**LPIPS** (↓)	0.1822	0.0966	0.0776	0.0827	0.0805	0.0758	**0.0741**
**GMSD** (↓)	0.2471	0.1655	0.1494	0.1514	0.1529	0.1489	**0.1479**

↑: higher values indicate better performance; ↓: lower values indicate better performance.

## Data Availability

This study used publicly available data from the NYU fastMRI Initiative (https://fastmri.med.nyu.edu/, accessed on 16 September 2025). The dataset is owned and maintained by New York University and NYU Langone Health and was accessed under the NYU fastMRI Dataset Sharing Agreement.

## Abbreviations

The following abbreviations are used in this manuscript:

CAIPI	Controlled Aliasing in Parallel Imaging
CS	Compressed Sensing
DC	Data Consistency
FSIM	Feature Similarity Index Measure
GRAPPA	Generalized Autocalibrating Partially Parallel Acquisition
HFEN	High-Frequency Error Norm
LPIPS	Learned Perceptual Image Patch Similarity
MoDL	Model-Based Deep Learning
MRI	Magnetic Resonance Imaging
NRMSE	Normalized Root Mean Square Error
PF	Partial Fourier
PI	Parallel Imaging
PSNR	Peak Signal-to-Noise Ratio
SENSE	Sensitivity Encoding
SSIM	Structural Similarity Index Measure
ZS-SSL	Zero-Shot Self-Supervised Learning
Zero-MIRID	Zero-shot Multi-shot Image Reconstruction for Improved Diffusion MRI
