# Correction: Cho, J. Logarithmic Scaling of Loss Functions for Enhanced Self-Supervised Accelerated MRI Reconstruction. *Diagnostics* 2025, *15*, 2993

**DOI:** 10.3390/diagnostics16040553

**Published:** 2026-02-13

**Authors:** Jaejin Cho

**Affiliations:** Department of Artificial Intelligence and Robotics, Sejong University, Seoul 05006, Republic of Korea; jaejincho@sejong.ac.kr

In the original publication [1], there was a mistake in Figure 2. Figure 2 in the published version is a duplicate of Figure 4 (with a different colormap), which is incorrect and may mislead readers. The corrected Figure 2 appears below. The authors state that the scientific conclusions are unaffected. This correction was approved by the Academic Editor. The original publication has also been updated.

## Figures and Tables

**Figure 2:**
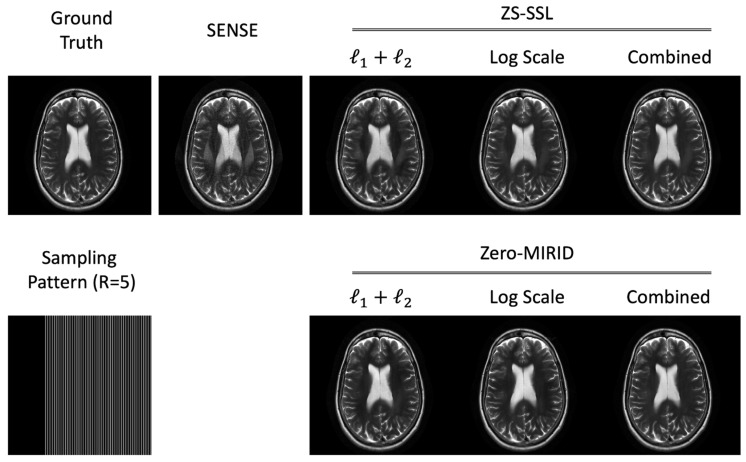
Reconstructed images from 1D subsampled data. The *k*-space data were retrospectively subsampled (*R* = 5) using a 6/8 PF acquisition, as illustrated in the sampling pattern.
